# Synthesis of Nitrogen Heterocycles Using Samarium(II) Iodide

**DOI:** 10.3390/molecules22112018

**Published:** 2017-11-21

**Authors:** Shicheng Shi, Michal Szostak

**Affiliations:** Department of Chemistry, Rutgers University, 73 Warren Street, Newark, NJ 07102, USA

**Keywords:** samarium iodide, nitrogen heterocycles, nitrogen, radicals, reductive coupling, SmI_2_, radical cyclizations, samarium diiodide, umpolung cyclizations, aminoketyl radicals

## Abstract

Nitrogen heterocycles represent vital structural motifs in biologically-active natural products and pharmaceuticals. As a result, the development of new, convenient and more efficient processes to *N*-heterocycles is of great interest to synthetic chemists. Samarium(II) iodide (SmI_2_, Kagan’s reagent) has been widely used to forge challenging C–C bonds through reductive coupling reactions. Historically, the use of SmI_2_ in organic synthesis has been focused on the construction of carbocycles and oxygen-containing motifs. Recently, significant advances have taken place in the use of SmI_2_ for the synthesis of nitrogen heterocycles, enabled in large part by the unique combination of high reducing power of this reagent (E_1/2_ of up to −2.8 V) with excellent chemoselectivity of the reductive umpolung cyclizations mediated by SmI_2_. In particular, radical cross-coupling reactions exploiting SmI_2_-induced selective generation of aminoketyl radicals have emerged as concise and efficient methods for constructing 2-azabicycles, pyrrolidines and complex polycyclic barbiturates. Moreover, a broad range of novel processes involving SmI_2_-promoted formation of aminyl radicals have been leveraged for the synthesis of complex nitrogen-containing molecular architectures by direct and tethered pathways. Applications to the synthesis of natural products have highlighted the generality of processes and the intermediates accessible with SmI_2_. In this review, recent advances involving the synthesis of nitrogen heterocycles using SmI_2_ are summarized, with a major focus on reductive coupling reactions that enable one-step construction of nitrogen-containing motifs in a highly efficient manner, while taking advantage of the spectacular selectivity of the venerable Kagan’s reagent.

## 1. Introduction

Since its introduction to organic synthesis by Kagan in 1980, samarium diiodide (SmI_2_, Kagan’s reagent) has, arguably, become the most useful single electron transfer reagent to effect polarity inversion in challenging transformations [1,2,3,4,5]. The synthetic utility of SmI_2_ is evident from the numerous applications in complex total syntheses [6,7] and large scale pharmaceutical manufacturing [8], where the combination of high redox potential (E_1/2_ of up to –2.8 V) [9] with excellent and unique chemoselectivity of SmI_2_ [10] enables a wide range of chemical transformations impossible to achieve with other single- or two-electron transfer reagents. The widespread adoption of SmI_2_ by organic chemists has been possible owing to several clear advantages of SmI_2_, including: (1) the ability to fine-tune the reactivity by inorganic, protic and Lewis basic additives [11,12]; (2) the capacity to trigger reductive cyclizations via complementary radical or anionic mechanisms [13]; (3) well-defined mechanistic manifold under typically thermodynamic control [14]; (4) rapid access to complex architectures with precise stereochemistry enabled by high Lewis acidity of Sm(II)/(III) [15]; and, most importantly, (5) the operational-simplicity of preparing and using SmI_2_ in a standard laboratory setting without the requirement for special equipment or reaction set-up [16].

Historically, the use of SmI_2_ in organic synthesis has been focused on the construction of carbocycles and oxygen-containing motifs [1,2,3,4,5,6,7]. Complex reductive cyclization processes forming carbocyclic skeletons relying on the selective generation of ketyl radicals have now become a routine part of our synthetic toolbox [1,2,3,4,5,17,18]. Great strides have been made in applying SmI_2_ to the assembly of stereodefined oxacycles by polarity inversion of oxygen-containing carbonyl electrophiles [19,20,21,22]. Moreover, recent elegant studies further established the potential of SmI_2_ in asymmetric synthesis of carbocycles [23].

**Figure 1:**
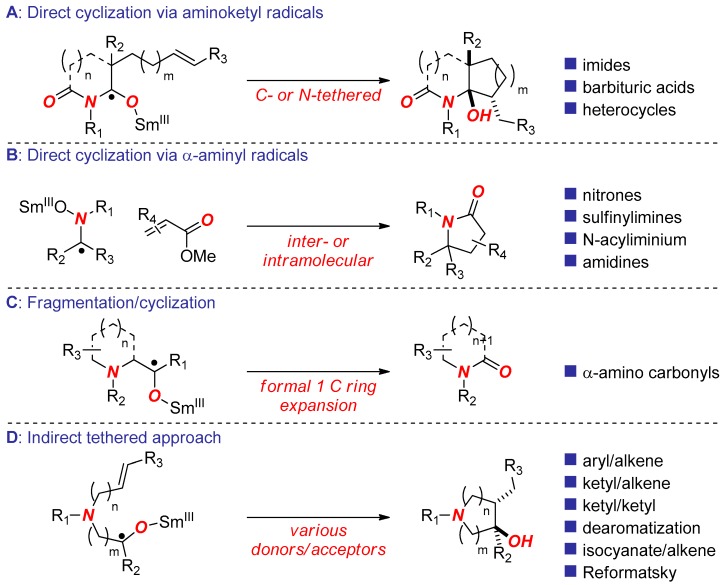
Approaches to the Synthesis of Nitrogen Heterocycles using Samarium(II) Iodide.

In this context, recently major advances have taken place in the use of SmI_2_ for the synthesis of nitrogen heterocycles (Figure 1). Nitrogen heterocycles represent vital structural motifs in biologically-active natural products and pharmaceuticals [24,25,26]. A plethora of nitrogen heterocycles have gained privileged status in medicinal chemistry [27]. However, the full potential of SmI_2_ in the synthesis of nitrogen-containing motifs is yet to be fully realized. This is likely due to two factors: (1) high Lewis basicity of nitrogen-containing functional groups, which may result in preferential coordination and displacement of ligands required for efficient electron transfer and cyclization steps using SmI_2_; and (2) high activation energy required for the direct electron transfer to nitrogen-containing carbonyl groups.

This review summarizes the current-state-of-the-art in the use of SmI_2_ for the synthesis of nitrogen heterocycles, including the literature through October 2017. The major focus is placed on reductive coupling reactions that enable one-step construction of nitrogen-containing motifs in a highly efficient manner. The selected examples serve to demonstrate the versatility offered by SmI_2_ and highlight the areas for further improvement. Therefore, the review is not comprehensive and only a selection of the most significant developments is presented.

The major focus has been placed on mechanistic pathways, selectivity and synthetic advantages of reductive coupling processes mediated by SmI_2_. The review is arranged by the type of reductive coupling method that has been utilized in the synthesis of N-heterocycles with SmI_2_ (Figure 1). At present, SmI_2_ can be employed to furnish nitrogen heterocycles by four general mechanisms: (1) direct generation of aminoketyl radicals; (2) cross-coupling of α-aminyl radicals; (3) fragmentation/cyclization; and (4) indirect tethering approach. The final section of the review summarizes recent advances in the generation of aminoketyl and related radicals. These reactions provide a proof-of-principle and direction in which SmI_2_ technology can expand the assembly of nitrogen heterocycles for broad synthetic applications. It is our hope that the review will provide a one-stop overview of this important topic and stimulate further progress in the synthesis of nitrogen heterocycles using the venerable Kagan’s reagent.

## 2. Synthesis of Nitrogen Heterocycles via Aminoketyl Radicals

Direct cyclization of aminoketyl radicals represents the most general method for the synthesis of nitrogen heterocycles with SmI_2_. However, in contrast to the broad utility of ketyl and α-aminyl radicals, the development of practical methods for the addition of aminoketyl radicals to unactivated π-acceptors has been challenging due to the prohibitive stability of the amide bond to electron transfer, resulting from n_N_ → π^*^_CO_ conjugation [28,29].

**Scheme 1:**
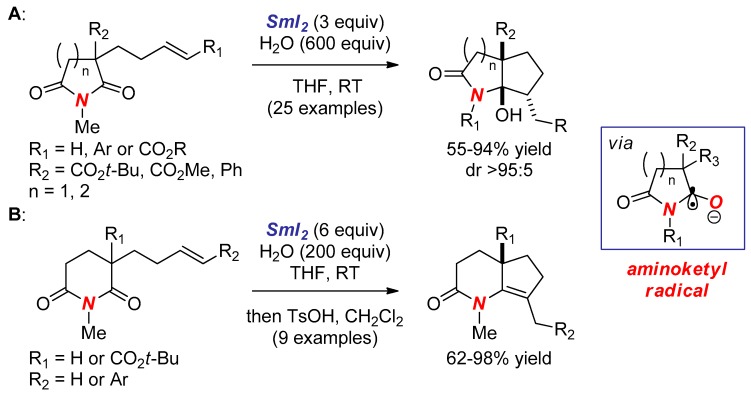
(**A**) Synthesis of 2-Azabicycles via Reductive Cyclization of Cyclic Imides; (**B**) Reductive Cyclization/Dehydration of Cyclic Imides.

In 2015, we have introduced the first general method for the generation of unactivated aminoketyl radicals and applied these precursors in the highly efficient cyclizations to afford 2-aza-bicycles containing up to three contiguous stereocenters with excellent stereoselectivity (Scheme 1A) [30]. The key to the successful development of this process relied on combining structural features of the amide bond in the imide template (low energy antibonding π^*^orbital, n_N_ → π^*^_CO_ delocalization into the remaining carbonyl, conformationally-locked system to prevent N–C_α_ fragmentation) with anomeric-type stabilization of the aminoketyl radical anion intermediate, facilitating electron transfer. The functional group tolerance is very broad, including halides (Br, Cl), esters, lactams, highly electron-deficient and sterically-hindered arenes. Both 5- and 6-membered imides undergo cyclization in high yields. Subsequently, a tandem, one-pot reductive cyclization/dehydration protocol was developed to conveniently access enamides featuring an endocyclic olefin for further functionalization (Scheme 1B) [31]. The advantage of using imides in cyclization is readily apparent. The highly selective SmI_2_–H_2_O system [12] can easily differentiate between three similar carbonyl groups, selectivity effecting SET to one of the imide carbonyls. The product 2-aza-bicycles are prominent features in a wide range of alkaloids, medicines and ligands (cf. less general products from stabilized barbituric acids). The process is scalable and the products are easy to isolate because the nitrogen is protected by the acyl group.

**Scheme 2:**
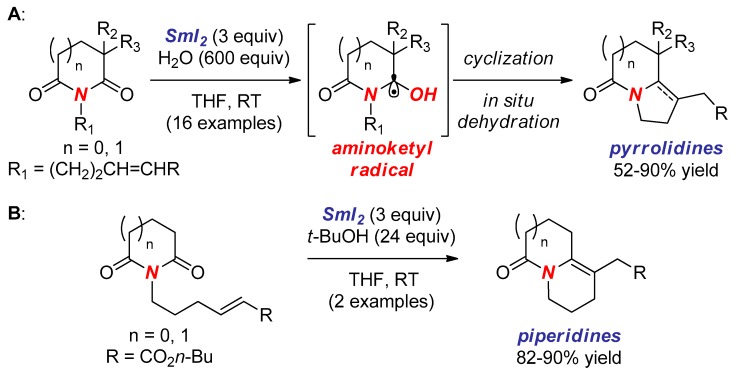
Synthesis of Pyrrolidines and Piperidines via Reductive Cyclization of N-Tethered Cyclic Imides: (**A**) Construction of Pyrrolidine Scaffolds; (**B**) Construction of Piperidine Scaffolds.

In 2016, we have reported direct cyclizations of aminoketyl radicals using N-tethered precursors (Scheme 2) [32]. While positioning of the π-acceptor tether at the α-position to the imide carbonyl group in a 1,3-arrangement enabled efficient reductive 5-exo cyclizations, likely facilitated by the presence of a directing group [33], the N-tethered cyclization is significantly more challenging due to geometrical constraints of the planar imide template. The reaction generates fused pyrrolidine or piperidine scaffolds containing up to four functional handles for further functionalization in 2–3 steps from commercial materials. The product indolizidine and quinazolidine lactams are of particular significance in medicinal chemistry and natural product synthesis. The protocol relies on the high reducing potential of the Kagan’s reagent to selectively transfer electrons to the unactivated imide carbonyl, clearly underscoring the advantage of using the selective SmI_2_–H_2_O system. Moreover, we found that the reduction of imides (e.g., glutarimide, E_1/2_ = −2.64 V vs. SCE in CH_3_CN) is favored over the model six-membered lactone (tetrahydro-2*H*-pyran-2-one, E_1/2_ = −2.96 V vs. SCE in CH_3_CN) [34], which suggests that a myriad of reductive cyclization processes is feasible in analogy to the elegant reductive cyclizations of lactones [19,20,21,22].

**Scheme 3:**
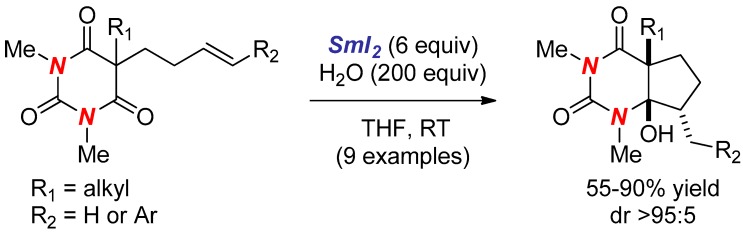
SmI_2_-Mediated Reductive Cyclizations of Barbituric Acids (Cyclic 1,3-Diimides) via Aminoketyl Radicals.

Reductive cyclizations of barbituric acid derivatives proceeding via aminoketyl radicals were reported by Szostak and Procter in 2013 (Scheme 3) [35]. The reaction constituted the first example of selective reductive umpolung cyclizations exploiting ketyl-type radicals generated from barbituric acids, and provided an efficient entry to functionalized pyrimidine scaffolds. Interestingly, all products were formed with excellent stereoselectivity as a result of increased stabilization of the aminoketyl radical in this scaffold. However, it should be clearly noted that the generality of the barbituric acid cyclizations is much lower than that of imides due to structural limitations of the cyclic 1,3-dimide template.

**Scheme 4:**
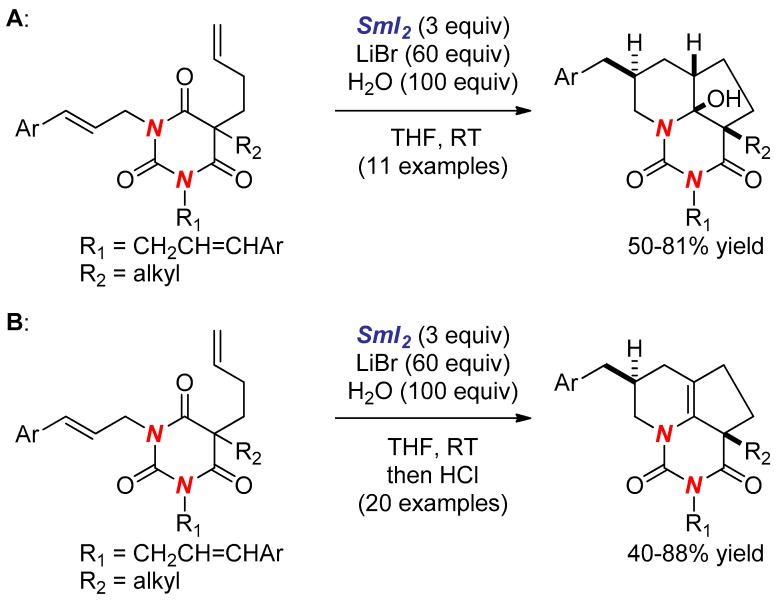
Synthesis of Polycyclic Barbiturates via Cascade Cyclizations: (**A**) Synthesis of Tricyclic Barbiturates; (**B**) Synthesis of Tricyclic Barbiturates by Cross-Coupling/Dehydration.

**Scheme 5:**
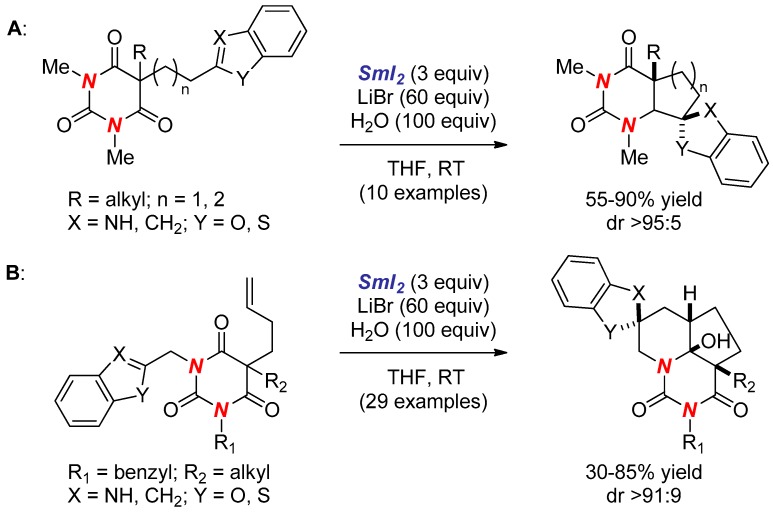
Synthesis of Spiro-Barbiturates via Dearomatizing Cyclizations: (**A**) Direct Cyclizations; (**B**) Cascade Cyclizations.

Concurrently to our studies on reductive couplings of cyclic imides, the Procter group elegantly demonstrated the synthetic potential of aminoketyl radicals stabilized by the barbiturate ring (Scheme 4 and Scheme 5) [36]. In the first generation approach, radical cascade cyclizations initiated by the selective electron transfer to the diimide carbonyl, followed by the addition of carbon-centered radical intermediates to the N-tethered π-acceptor were developed (Scheme 4). This mechanistically distinct process from direct cyclizations of aminoketyl radicals onto N-tethered acceptors (see Scheme 2) provided the first proof-of-principle evidence for reductive cascade cyclizations of aminoketyl radicals, thus generating complex nitrogen heterocycles. Importantly, the authors demonstrated that by fine-tuning the reaction conditions it is possible to selectively furnish hemiaminal products (Scheme 4A) or dehydrated enamides (Scheme 4B). The process employed a rarely utilized SmI_2_–LiBr–H_2_O reagent system [1,2], which may promote the second radical cyclization by increasing the redox potential of the SmI_2_–H_2_O reagent. The steric bulk of SmBr_2_–H_2_O may also result in the slower outer-sphere process. In addition to generating up to five new stereocenters with excellent stereoselectivity (up to >95:5 dr), rapid formation of novel tricyclic pyrimidine-like scaffolds is an added benefit of this protocol.

Subsequently, the Procter group also reported stereoselective dearomatizing cyclizations of barbituric acids via aminoketyl radicals (Scheme 5) [37]. Mechanistically, this process involves direct addition of the aminoketyl radical stabilized by the barbiturate ring onto C-tethered benzofused aromatic ring (benzofuran or benzothiazole) or a cascade cyclization of the C-tethered π-acceptor, followed by the addition of carbon-centered radical onto the benzofused aromatic ring (benzofuran, benzothiazole, benzoxazole, benzothiophene, naphthalene). Impressive functional group tolerance has been demonstrated, including aryl halides, ethers and heterocycles. This elegant process sets the stage for the design of a plethora of dearomatizing cyclizations for the synthesis of nitrogen heterocycles via aminoketyl radicals [38,39].

**Scheme 6:**
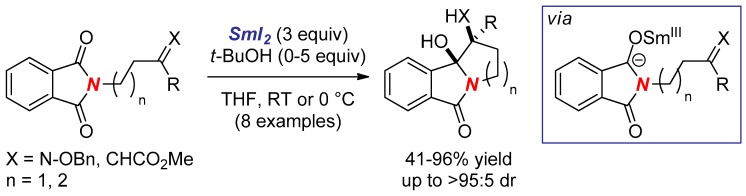
Reductive Cyclizations of N-Substituted Phthalimides via Anionic Coupling.

The successful construction of nitrogen heterocycles via aminoketyl radicals depends on the capacity of the Sm(II) reagent to generate and stabilize the formed radical to prevent reduction to the anion. In an alternative mechanism, Chiara reported the SmI_2_-mediated reductive cross-coupling between phthalimides and activated olefins, nitrones, and oxime ethers (Scheme 6) [40]. The reaction affords α-hydroxy lactams in high yields and with generally good stereoselectivity. Mechanistically, the method involves reduction of N-tethered phthalimide (E_1/2_ = −1.49 V vs. SCE in CH_3_CN) [32] to the anion, followed by anionic addition. In this case, the reactivity is limited to phthalimides, wherein the benzylic position facilitates the electron transfer and stabilizes the formed anion.

**Scheme 7:**
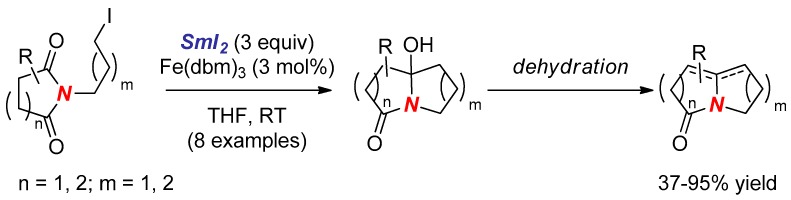
Synthesis of Lactams via Ionic Cyclization of N-Tethered Iodoalkyl Cyclic Imides.

In a synthetically related development, the Ha group developed reductive cyclizations of N-iodoalkyl tethered cyclic imides using the SmI_2_/Fe(dbm)_3_ reagent system (Scheme 7) [41,42]. The reaction affords bicyclic lactams via nucleophilic addition of the organosamarium; however, a limitation of this protocol is the generation of isomeric olefin products.

## 3. Synthesis of Nitrogen Heterocycles via Aminyl Radicals

SmI_2_-mediated cross-coupling of imines and equivalents via α-aminoalkyl radicals is well-established [1,2]. Broadly speaking, formation of α-aminoalkyl radicals using SmI_2_ is generally much easier than aminoketyl radicals owing to the higher reactivity of precursors [43], which could potentially lead to wide applications in organic synthesis. However, despite significant progress in the last 15 years, protocols for the chemoselective cross-coupling of imines and equivalents via α-aminoalkyl radicals are yet to reach the level of utility of their ketyl counterparts.

**Scheme 8:**
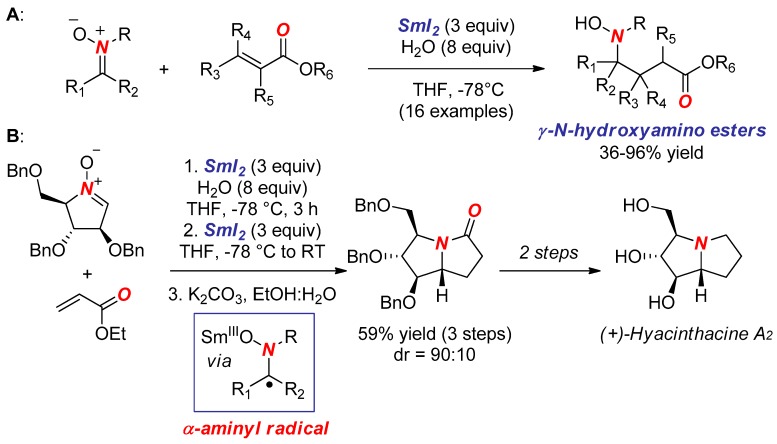
(**A**) SmI_2_-Promoted Cross-Coupling of Nitrones with α,β-Unsaturated Esters via Aminyl Radicals; (**B**) Synthesis of (+)-Hyacinthacine A_2_ by Cross-Coupling of Cyclic Nitrones.

Seminal studies by Py and Vallée showed the feasibility of polarity reversal of C=N bonds in nitrones in the cross-coupling with ketones and aldehydes [44]. Mechanistic studies demonstrated direct electron transfer to the nitrone group, resulting in the formation of an α-aminoalkyl radical, followed by addition to the carbonyl group. In 2003, another major breakthrough was reported by Py and Vallée in the chemoselective conjugate additions of nitrones to α,β-unsaturated esters (Scheme 8A) [45,46]. The reaction generates γ-N-hydroxyamino esters, which could be readily converted into the corresponding pyrrolidines upon deoxygenation and base-induced cyclization. At the same time, similar studies were reported by Skrydstrup [47,48]. Owing to the high stability of nitrones, ease of synthesis and high efficiency in polarity reversal using SmI_2_, nitrones are among the most versatile precursors to α-aminoalkyl radicals, while their reactivity compares favorably with oximes, oxime ethers, hydrazones, sulfonyl imines and N-acyliminiums [1,2,43].

**Scheme 9:**
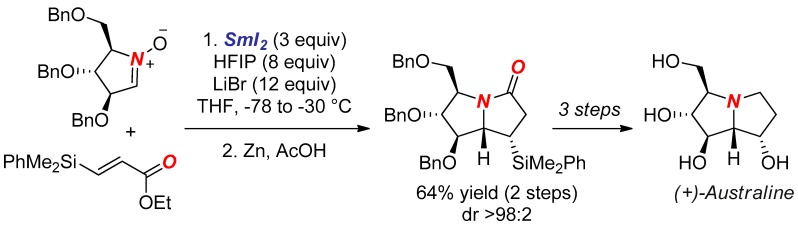
Synthesis of (+)-Australine by Cross-Coupling Cyclic Nitrones with β-Silyl Acrylates.

Py and co-workers developed the cross-coupling of nitrones with α,β-unsaturated acceptors as an attractive methodology for the synthesis of γ-lactams [49,50] and pyrrolizidine alkaloids [51,52,53]. In 2005, they reported the total synthesis of (+)-hyacinthacine A_2_, a polyhydroxylated amyloglucosidase inhibitor, using SmI_2_-mediated reductive coupling between a chiral l-xylose-derived cyclic nitrone and ethyl acrylate to generate the key bicyclic ring system (Scheme 8B) [52]. Mild reaction conditions, selective cross-coupling/deoxygenation and the synthesis of densely functionalized pyrrolizidine alkaloid scaffold are noteworthy. The cross-coupling approach was further highlighted by the Py group in the synthesis of (+)-australine (Scheme 9) [53]. Notably, readily available β-silyl acrylates with silicon serving as an oxygen equivalent were demonstrated as highly viable alternatives to β-alkoxy acrylates. An interesting feature of this protocol involves the use of both water and LiBr as SmI_2_ additives to increase the redox potential of the reagent and stereoselectivity of the process.

**Scheme 10:**
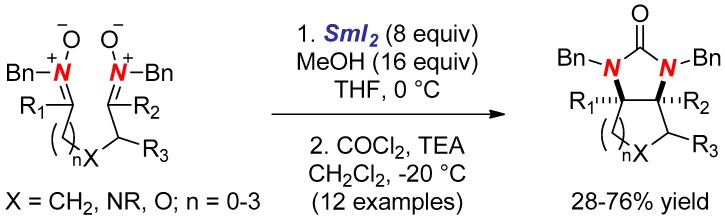
Synthesis of Cyclic Ureas by Intramolecular Pinacol-Coupling of Dinitrones.

Intramolecular cross-coupling of nitrones is also feasible. In 2005, Skrydstrup and co-workers demonstrated the synthesis of cyclic ureas by SmI_2_-mediated intramolecular pinacol-type coupling of dinitrones (Scheme 10) [54]. The reaction forms *cis*-diamines in a highly diastereoselective manner. The authors found that proton donors have a significant impact on the efficiency and stereoselectivity of the coupling with MeOH providing the optimum results. This reaction is an interesting alternative to well-established methods for the synthesis of cyclic ureas [55].

**Scheme 11:**
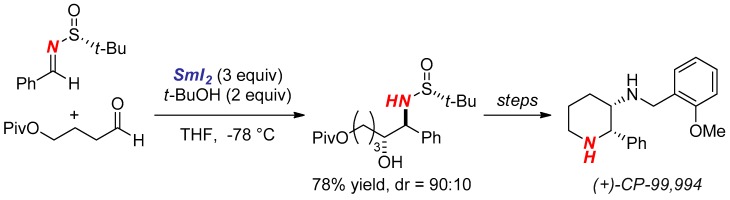
Synthesis of (+)-CP-99,994 by Cross-Coupling of *N*-*tert*-Butanesulfinyl Imines.

The use of *N*-*tert*-butanesulfinyl imines [56] as precursors to α-aminoalkyl radicals is also promising. In 2005, in a striking development, Xu and Lin demonstrated the first SmI_2_-mediated intermolecular cross-coupling of *N*-*tert*-butanesulfinyl imines with aldehydes. The reaction affords β-amino alcohols in excellent diastereo- and enantioselectivity [57]. The generation of chiral α-aminoalkyl radicals or highly nucleophilic aza-anions [58,59] provides novel opportunities for the synthesis of nitrogen heterocycles using Ellman’s *N*-*tert*-butanesulfinyl imines as the chirality source. The selective SmI_2_-promoted formation of chiral β-amino alcohols has been highlighted in the synthesis of NK-1 SP receptor antagonist, (+)-CP-99,994 (Scheme 11) [60].

**Scheme 12:**
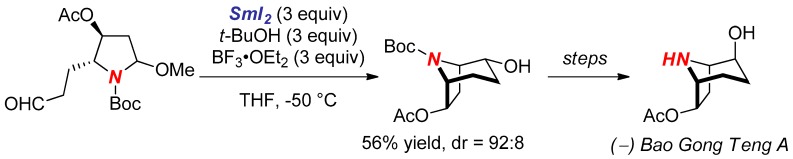
Synthesis of (−)-Bao Gong Teng A by Cross-Coupling of N,O-Acetals.

**Scheme 13:**
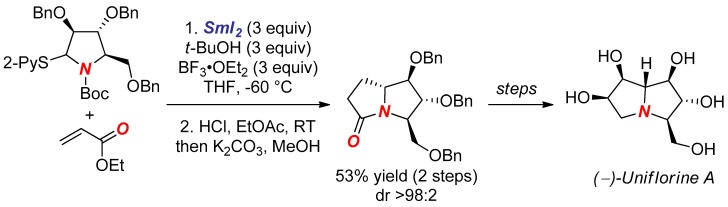
Synthesis of (−)-Uniflorine by Intermolecular Cross-Coupling of *N*,*S*-Acetals.

The reduction of *N*-acyliminium ions [61] with SmI_2_ represents another method to generate α-aminoalkyl radicals for the construction of nitrogen heterocycles. In particular, this method offers advanatges in terms of improved reaction efficiency and selectivity using cyclic N-acyliminium precursors. In 2011, Huang and co-workers reported the synthesis of a hydroxylated tropane alkaloid, (−)-bao gong teng A, by the intramolecular *N*,*O*-acetal/aldehyde coupling (Scheme 12) [62]. Mechanistically, the reaction involves BF_3_-promoted generation of the N-acyliminium followed by SET to generated α-aminyl radical. The authors proposed that the preferential formation of the equatorial alcohol (dr = 92:8) results from repulsive electronic interactions between N and O lone pairs in the transition state. In cases when higher reactivity is required, *N*,*S*-acetals provide advantageous results. This concept was nicely demonstrated by Huang and co-workers in the synthesis of (−)-uniflorine by intermolecular acetal/α,β-unsturated ester cross-coupling as a key step (Scheme 13) [63]. Mechanistic studies demonstrated that in the presence of BF_3_·Et_2_O and *t*-BuOH, the reaction proceeds via a radical (cf. anionic) pathway. The reductive coupling product was readily converted to the pyrrolizidone by Boc removal and K_2_CO_3_-promoted cyclization.

**Scheme 14:**
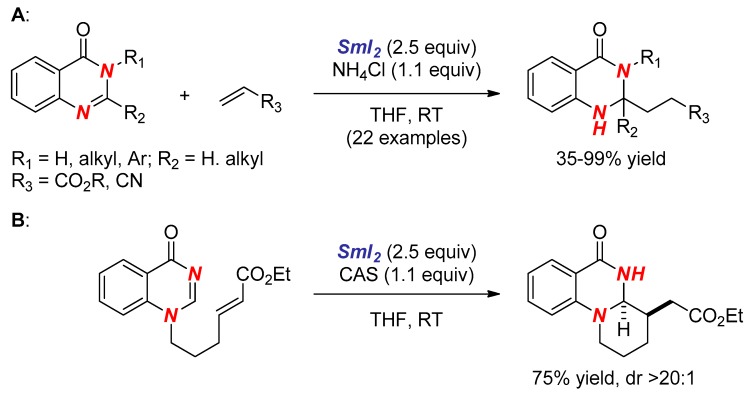
(**A**) SmI_2_-Promoted Intermolecular Cross-Coupling of Amidines; (**B**) Synthesis of Bicyclic Aminals via Intramolecular Cross-Coupling.

An interesting strategy to generate aminal radicals for the synthesis of nitrogen heterocycles was recently reported by Beaudry and co-workers (Scheme 14) [64,65]. Here, the required aminal radicals were generated from the corresponding amidines using a novel SmI_2_–NH_4_Cl system. In some cases, CSA (camphorosulfonic acid) in place of NH_4_Cl was shown to give higher reaction efficiency. The scope of the reaction is very broad, including intermolecular cross-couplings of various benzene-fused (quinazolinones), aliphatic and spirocyclic amidines with α,β-unsaturated esters and acrylonitrile (Scheme 14A). Two examples of intramolecular cyclizations using N-tethered olefin acceptors were also reported, and proceeded with excellent diastereoselectivity (Scheme 14B). The methodology was further expanded to the use of amidinium ions as precursors to aminal radicals. Mechanistic studies demonstrated that the reaction involves SET to the amidine substrate to afford aminal radical, followed by addition to the π-acceptor. Importantly, the SmI_2_-mediated process provides synthetic advantages in terms of mild reaction conditions, decreased waste generation and operational simplicity over the AIBN/Bu_3_SnH-promoted radical translocation method reported earlier by the same authors [66].

## 4. Synthesis of Nitrogen Heterocycles via Fragmentation/Cyclization Pathways

**Scheme 15:**
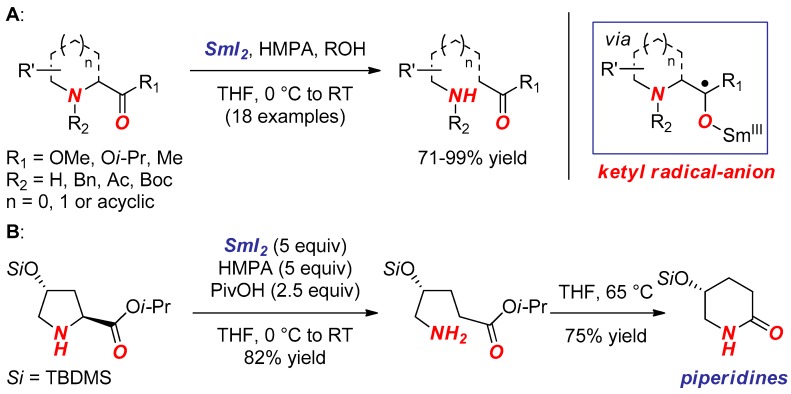
(**A**) SmI_2_-Promoted Reductive Deamination of α-Amino Esters and Ketones; (**B**) Synthesis of Chiral Piperidines by Fragmentation/Cyclization Pathway.

Another pathway for the synthesis of nitrogen heterocycles with SmI_2_ involves chemoselective cleavage of C–N bonds of α-aminocarbonyl compounds, followed by ionic cyclization (Scheme 15) [66,67,68]. Honda reported that α-amino esters and ketones undergo selective scission of the C–N bond upon exposure to the SmI_2_–HMPA–ROH system [67]. Although simple phenylalanine derivatives undergo efficient deamination, the synthetic value of this method hinges upon the use of cyclic proline and pipecoline derivatives, which afford γ- and δ-amino acids (Scheme 15A). The chemoselectivity of this method is high, with overreduction of the ketone or ester group not observed under the mild SmI_2_–HMPA conditions. The temperature-induced intramolecular cyclization of the chiral amino ester products was elegantly applied in the synthesis of piperidine derivatives (Scheme 15B) [68,69].

**Scheme 16:**
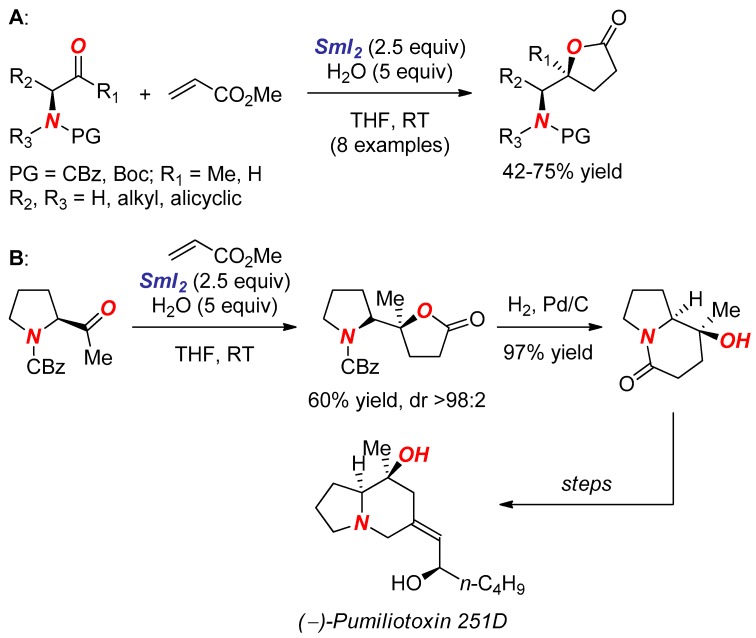
(**A**) SmI_2_-Promoted Intermolecular Cross-Coupling of α-Amino Acids; (**B**) Synthesis of (−)-Pumiliotoxin 251D.

Interestingly, Burtoloso recently engaged a related group of α-aminocarbonyl substrates in the intermolecular cross-coupling with methyl acrylate to form γ-aminomethyl-γ-butyrolactones using SmI_2_/H_2_O (Scheme 16A) [70]. The reaction proceeds in high yields and with excellent diastereoselectivity. Importantly, cleavage of the N–C bond was not observed, which likely results from the complementary Sm(II) reagent system employed. This transformation, which rapidly delivers chiral β-amino alcohol units, represents a powerful method for the construction of piperidine, indolizidine and quinolizidine alkaloids from readily available α-amino acid derivatives (Scheme 16B) [71,72].

## 5. Synthesis of Nitrogen Heterocycles via Tethered Approach

The SmI_2_-mediated synthesis of nitrogen heterocycles by an indirect tethered approach, wherein the nitrogen atom is not directly involved in radical or ionic cross-coupling represents a common and popular strategy in organic synthesis. In general, nitrogen heterocycles are formed selectively by several complementary mechanisms exploiting the reductive and coordinating properties of SmI_2_, including (1) aryl radical/alkene cross-coupling; (2) ketyl radical/alkene cross-coupling; (3) pinacol-type couplings; (4) dearomatizing ketyl radical/arene cross-coupling; (5) olefin/isocyanate or carbodiimide cross-coupling; and (6) ionic Reformatsky-type reactions. In principle, the synthesis of nitrogen heterocycles by other radical or ionic mechanisms enabled by SmI_2_ is also possible, but these methods have not received much attention.

**Scheme 17:**
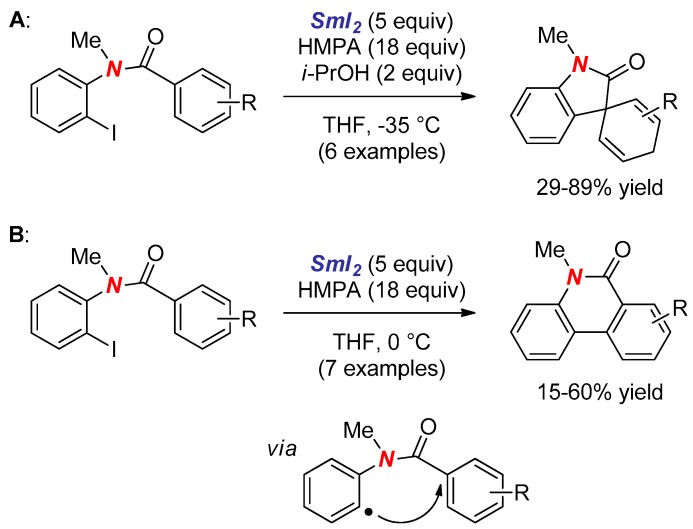
Synthesis of Spirocyclic Oxindoles (**A**) and 6-(5*H*)-Phenanthridinones (**B**) by Aryl Radical/Arene Cross-Coupling.

Tanaka reported an efficient intramolecular arylation of 2-iodo-benazanilides for the synthesis of spirocyclic oxindoles and 6-(5*H*)-phenanthridinones (Scheme 17) [73]. The reaction was initially conducted using the SmI_2_–HMPA system in the absence of protic additives, leading to selective formation of fused phenanthridinones. When the reaction was performed with 2.0 equivalents of *i*-PrOH, spirocyclic oxindoles products were obtained selectively in good yields. The mechanism was proposed to involve the following steps: (1) generation of the aryl radical; (2) 5-exo-trig cyclization to the spirocyclic radical intermediate; (3) protonation to give the spirocyclic oxindole product or rearrangement of the unstable spirocyclic radical to phenanthridinones.

**Scheme 18:**
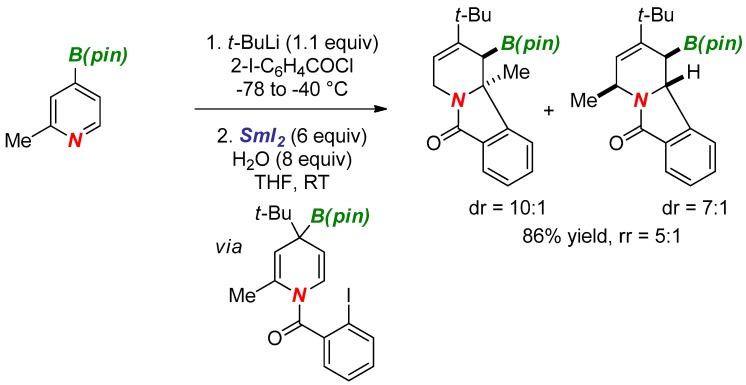
Synthesis of Dihydropyridine Boronate Esters by Aryl Radical/Alkene Cross-Coupling.

An interesting example of the SmI_2_-promoted aryl radical/alkene cyclization was recently reported by Ready and co-workers in their studies on nucleophilic addition of organometallic reagents to pyridine boronic esters (Scheme 18) [74]. After initial dearomatization of the pyridine ring, the reductive cyclization of a tethered aryl iodide with the SmI_2_–H_2_O reagent was used to generate the fused pyrrolidine ring system. The radical cyclization was accompanied by a 1,2-boron migration and olefin transposition forming versatile allyl boronic esters. The mechanism was proposed to involve 5-exo-trig cyclization, followed by B(pin) migration; however, additional studies are required to elucidate the mechanism. The method highlights the potential of SmI_2_ to provide attractive N-heterocyclic building blocks and products.

**Scheme 19:**
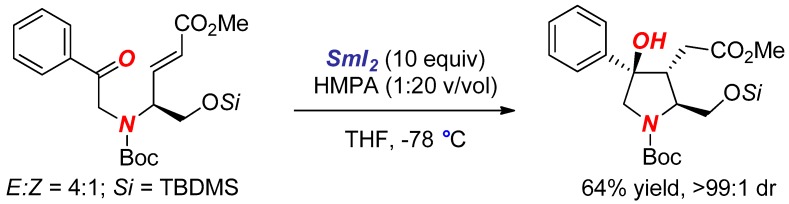
Synthesis of Kainoid Amino Acids by Ketyl Radical/Alkene Cross-Coupling.

The ketyl/alkene cross-coupling reported by Shirahama and co-workers is another illustration of the synthesis of pyrrolidines using SmI_2_ (Scheme 19) [75]. This process used SmI_2_–HMPA to form *trans*-substituted heterocycles, while in the presence of a protic additive, MeOH, *cis*-pyrrolidines were formed selectively. This was explained on the basis of a thermodynamic preference to adopt *trans*-conformation by minimizing steric repulsion between the samarium(III) alkoxide and methoxycarbonyl groups during the reversible electron transfer/cyclization steps.

**Scheme 20:**
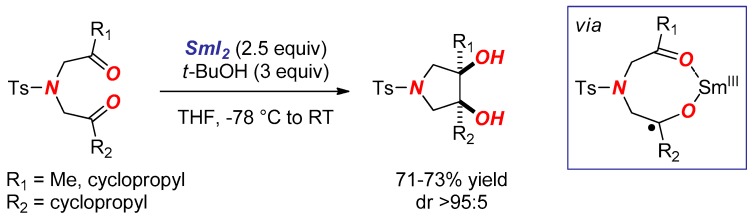
Synthesis of Cyclopropyl Pyrrolidines by Pinacol Coupling by Handa.

Carbonyl compounds (pinacol-type coupling) could be utilized in place of the electron-deficient π-acceptor to generate nitrogen heterocycles (Scheme 20) [76]. Using cyclopropyl radical clocks, Handa and co-workers demonstrated that the mechanism of SmI_2_-mediated ketone-ketone pinacol coupling in the synthesis of pyrrolidines likely involves the cyclization of a ketyl radical anion. The method is particularly useful for the synthesis of substituted pyrrolidine vicinal *cis*-diols with high diastereoselectivity.

**Scheme 21:**
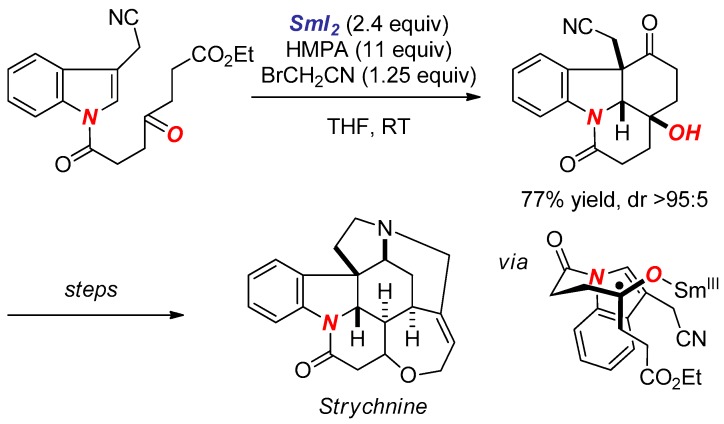
Intramolecular Ketone/Indole Dearomatizing Cross-Coupling: Synthesis of Strychnine.

Forming nitrogen heterocycles by SmI_2_-promoted dearomatization of readily available aromatics is attractive because of the potential to build-up of molecular complexity for the synthesis of alkaloids, high diastereoselectivity of the SmI_2_-mediated processes and the capacity of radical intermediates to participate in complex radical-anionic cascade transformations. Ketyl/indole dearomatizing cross-coupling have been pioneered by the Reissig group [77,78]. The synthetic utility of this method has been showcased in the total synthesis of strychnine (Scheme 21) [79,80,81]. The key reaction involves a SmI_2_–HMPA-mediated intramolecular 6-exo-trig ketyl/indole radical addition, followed by reduction and intramolecular acylation, furnishing the tetracyclic intermediate in 77% yield as a single diastereoisomer. Quenching the reaction with bromoacetonitrile improved the overall yield due to the undesired C–C fragmentation and loss of acetonitrile under the reaction conditions.

**Scheme 22:**
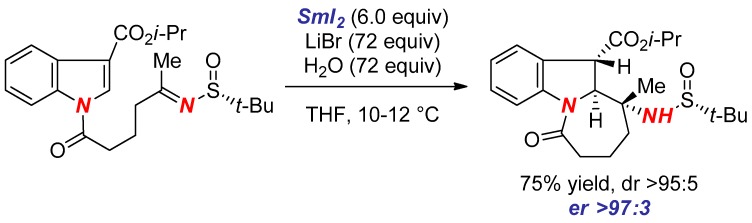
Intramolecular Sulfinyl Imine/Indole Dearomatizing Cross-Coupling.

More recently, the Reissig group extended their SmI_2_-mediated dearomatizing cross-coupling methodology to the intramolecular addition of sulfinyl imines to indoles (Scheme 22) [82]. Under the optimized conditions (SmI_2_–H_2_O–LiBr), sulfinyl imines undergo addition to the indole ring in good yields and modest to high diastereoselectivity. The preparation of enantiopure tertiary amines has been demonstrated; however, it should be noted that at present the major limitation of this method is reductive N–S cleavage prior to cyclization and substrate-dependent diastereoselectivity.

**Scheme 23:**
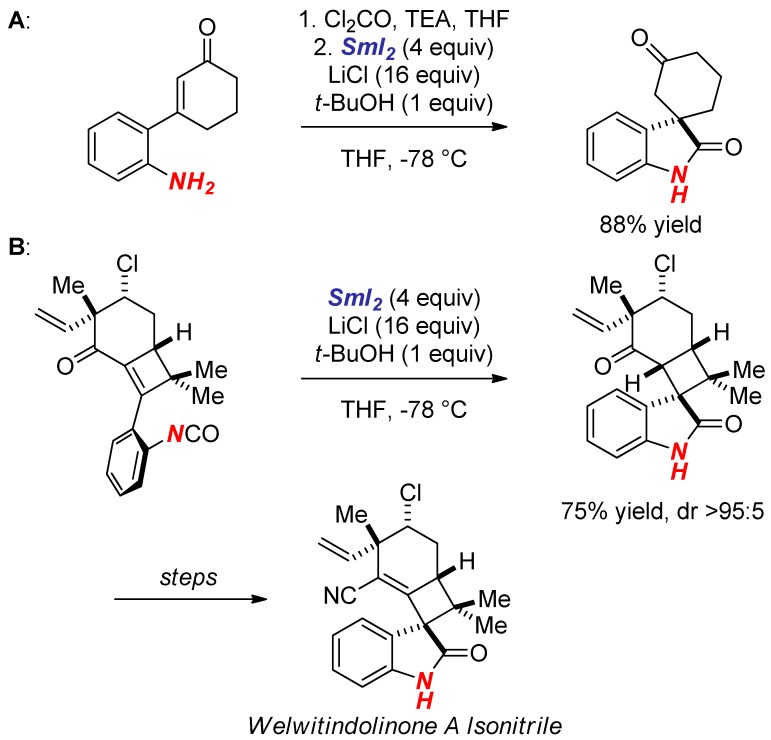
(**A**) Synthesis of Spirocyclic Oxindoles by Olefin/Isocyanate Cross-Coupling; (**B**) Application in the Synthesis of Welwitindolinone A Isonitrile.

Nitrogen heterocycles can be obtained via SmI_2_-mediated cross-coupling of stabilized radicals generated from activated π-acceptors with heterocumulenes, such as isocyanates and carbodiimides. In an impressive development, Wood and co-workers reported intramolecular cross-coupling of enones with isocyanates to afford spiro-oxindoles under very mild conditions (Scheme 23A) [83]. The SmI_2_–LiCl–*t*-BuOH system was found to give optimal performance in this reaction, likely due to increasing redox potential of Sm(II). The methodology was showcased in the total synthesis of welwitindolinone A isonitrile (Scheme 23B) [84]. The high chemoselectivity of this process, tolerating several sensitive functional groups, mild reaction conditions and full control of diastereoselectivity are particularly noteworthy.

**Scheme 24:**
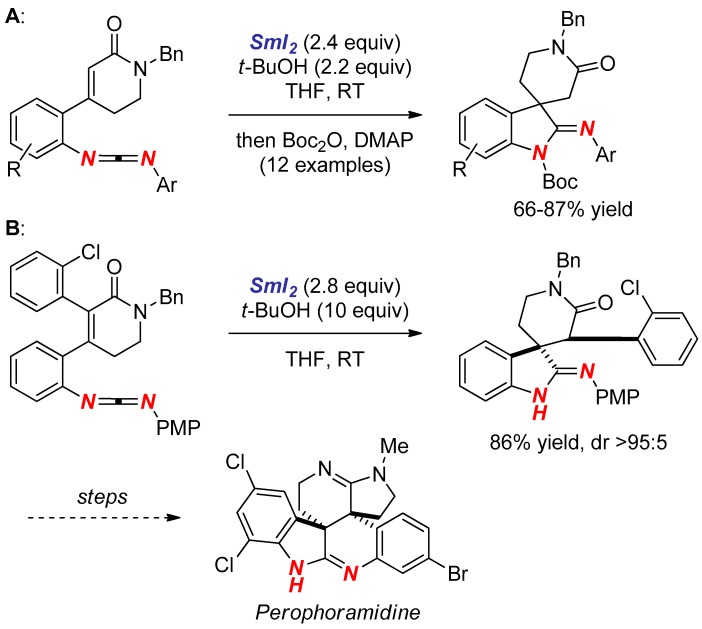
(**A**) Synthesis of Spirocyclic Amidines by Olefin/Carbodiimide Cross-Coupling; (**B**) Application in an Approach to Perophoramidine.

In a mechanistically related process, Takemoto reported the SmI_2_-mediated intramolecular cross-coupling of α,β-unsaturated amides with carbodiimides to give spirocyclic amidines (Scheme 24A) [85]. In the model study, they found that SmI_2_–*t*-BuOH system provided the highest yields. Subsequently, the reaction was utilized in the synthesis of a core system of perophoramidine (Scheme 24B) [86]. This very challenging cyclization involving SET reduction of a sterically-hindered tetrasubstituted olefin proceeded smoothly in the presence of SmI_2_–HMPA–*t*-BuOH at room temperature. The reaction gave a highly-functionalized spiro-2-iminoindoline ring system as a single diastereoisomer in 86% yield.

**Scheme 25:**
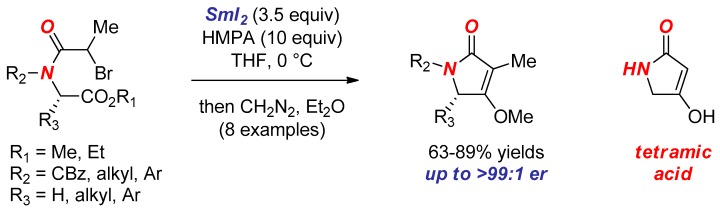
Synthesis of Tetramic Acids by Intramolecular Amide Reformatsky Cyclization.

In addition to reactions involving cross-coupling of radical intermediates, convenient methods for the preparation of nitrogen heterocycles via SmI_2_-mediated anionic coupling have been developed [13]. In particular, intramolecular Reformatsky reactions of α-halo amides have emerged as an important method to prepare nitrogen heterocycles. For example, Pettus demonstrated a general method for the synthesis of 3-methyl tetramic acids by cyclizing α-bromo amides into esters using SmI_2_–HMPA (Scheme 25) [87]. A variety of chiral α-bromo amides provided good yields of the tetramic acid products with excellent diastereocontrol. Importantly, racemization of the chiral stereocenter was not observed, highlighting the mild conditions of the SmI_2_-mediated protocol.

## 6. Reactions Involving Aminoketyl and Related Radicals

As outlined in the previous sections of this review, direct cyclizations of aminoketyl and related radicals provide one of the most efficient methods for the synthesis of nitrogen heterocycles. In this regard, recently significant advances have been made in the generation of simple, unfunctionalized aminoketyl and related radicals. These methods provide a proof-of-concept demonstration and direction in which SmI_2_-mediated electron transfer reactions can be used to expand the portfolio of nitrogen heterocycles for broad synthetic applications.

**Scheme 26:**
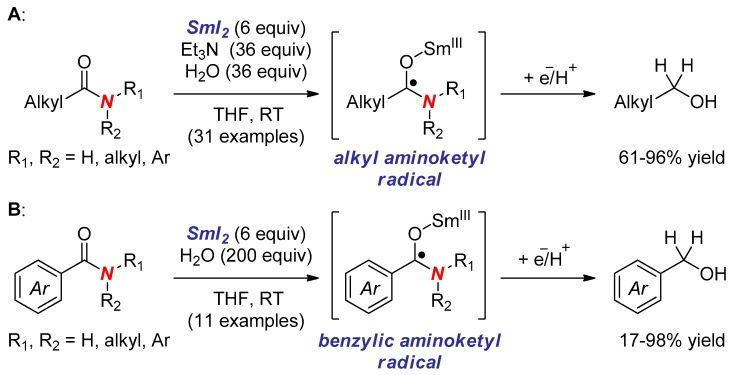
SmI_2_-Promoted Reduction of Amides via Aminoketyl Radicals: (**A**) Reduction of Alkyl Amides with SmI_2_/H_2_O/Et_3_N; (**B**) Reduction of Aromatic Amides with SmI_2_/H_2_O.

The reduction of amides by electron transfer mechanism represents a major challenge as a result of N_lp_ → π*_CO_ conjugation. In 2013, Szostak and Procter demonstrated the first reduction of aliphatic amides using SmI_2_–H_2_O–Et_3_N (Scheme 26A) [88]. The method is noteworthy due to the exquisite selectivity for the C–O vs. the more commonly observed N–C scission of the carbinolamine intermediate, resulting in a practical method for the reduction of all types of amides to the corresponding alcohols under mild conditions. More importantly, the optimized, highly reducing Sm(II) reagent system (E_1/2_ of up to –2.8 V) [89], relying on cooperative Lewis-base/proton donor coordination [90], enables generation of aminoketyl radicals from simple amides.

In 2017, we have demonstrated that both mild SmI_2_–H_2_O (E_1/2_ = –1.3 V vs. SCE) and more reducing SmI_2_–H_2_O–amine systems can be employed to reduce all types of benzamides with excellent N–C/C–O scission selectivity (Scheme 26B) [91]. In this case, generation of the aminoketyl radical is more facile by virtue of weakened amidic resonance, while the formed benzylic radicals show significantly higher stability due to delocalization. This bodes well for the development of reductive umpolung cyclizations via benzylic aminoketyl radicals as a key step.

**Scheme 27:**
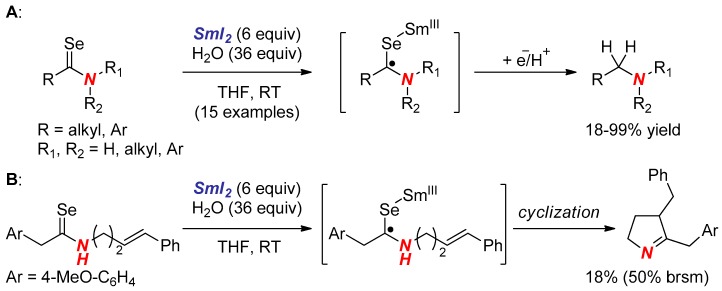
(**A**) SmI_2_-Promoted Reduction of Selenoamides; (**B**) Intramolecular Cycylization of Selenoamides via Aminyl-Type Radical with SmI_2_/H_2_O.

Another promising alternative was demonstrated by Procter and co-workers in the reduction of selenoamides using SmI_2_–H_2_O (Scheme 27A) [92]. They found that these precursors are selectively reduced to the corresponding amines under mild conditions. Moreover, an example of reductive cyclization of the formed aminoketyl-type radical onto an unactivated π-acceptor was demonstrated (Scheme 27B). The higher propensity of the selenoamide bond to reduction can be the basis for the development of selective cyclization cascades in the synthesis of nitrogen heterocycles.

**Scheme 28:**
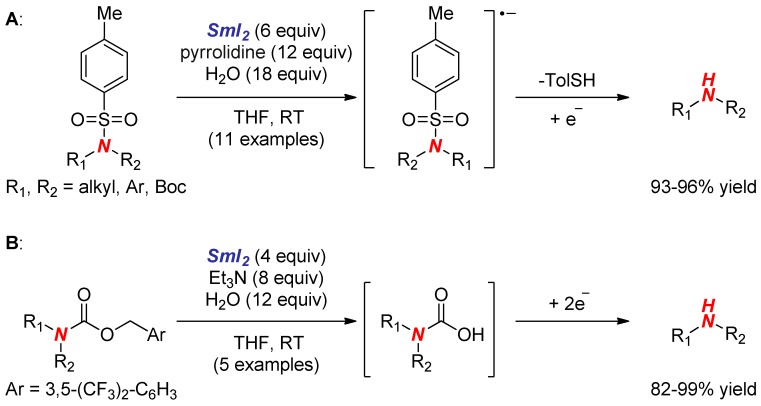
(**A**) Reductive Cleavage of Aryl Sulfonamides with SmI_2_/H_2_O/pyrrolidine; (**B**) Reductive Cleavage of Carbamates with SmI_2_/H_2_O/Et_3_N.

Furthermore, selective generation of nitrogen-centered radicals in the course of reduction of aryl sulfonamides via N–S scission (Scheme 28A) [93] and aminoketyl-type radicals during reductive C–O cleavage of a carbamate protecting group (CBTFB, 3,5-bis(trifluoromethyl)benzyloxycarbonyl) (Scheme 28B) [94] using SmI_2_–H_2_O–amine systems developed by Hilmersson should also be noted in this context. The first of these processes involves electron transfer to the sulfone aromatic ring, followed by fragmentation. Importantly, the reaction is fully selective for the reduction of aromatic sulfonamides (cf. aliphatic). The latter process involves C–O scission at the activated benzylic position [95,96], followed by two additional electron transfer events, and is highly selective for CBTFB cleavage in presence of other electrophilic groups, including *t*-Boc, Bn, and CBz.

## 7. Conclusions and Outlook

In conclusion, recently significant advances in the synthesis of nitrogen heterocycles using samarium(II) iodide have been achieved. These reactions have been enabled by the precise control of electron transfer events mediated by the strong reductant SmI_2_ in combination with the excellent chemoselectivity of the reductive cyclization steps. High reducing potential of SmI_2_ that can be rationally tuned by readily accessible ligands and additives, operational-simplicity of the processes mediated by SmI_2_, excellent functional group tolerance and exquisite diastereoselectivity, in particular in complex cascades, triggered by the coordinating ability of Sm(II)/(III) are among the major advantages of this reagent in the construction of nitrogen heterocycles. Importantly, as demonstrated in this review, the synthetic routes enabled by SmI_2_ are often inaccessible by other methods, highlighting the practical importance of SmI_2_ in organic synthesis.

The major recent developments include selective generation of aminoketyl radicals by direct electron transfer to the amide carbonyl group, efficient methods for the synthesis of complex heterocycles using α-aminoalkyl radicals, and the synthesis of nitrogen-containing molecular architectures by direct and tethered pathways. In addition, applications to the synthesis of natural products have highlighted the generality of nitrogen heterocycles accessible with SmI_2_.

Despite the significant progress, future research will need to address: (1) the synthesis of nitrogen heterocycles using SmI_2_ still lacks the generality of the construction of carbocyclic and oxygenated motifs; (2) it remains to be seen if the developed methods can be translated into the target synthesis of valuable products; (3) recent studies in asymmetric SmI_2_-mediated processes provide ample opportunities to apply this reactivity platform to the synthesis of nitrogen heterocycles, including by an indirect approach; (4) development of catalytic systems based on Sm(II) is indispensable to accelerate future research by reductive cross-coupling using lanthanides.

Given the recent advances and the vital role of nitrogen heterocycles in organic synthesis and medicinal chemistry, we are convinced that SmI_2_ will serve as a valuable springboard for this area of research.
